# Internet Claims on the Health Benefits of Cannabis Use

**DOI:** 10.1007/s11606-020-06421-w

**Published:** 2021-03-19

**Authors:** Nicholas Lau, Madeleine Gerson, Deborah Korenstein, Salomeh Keyhani

**Affiliations:** 1 San Francisco VA Medical Center, San Francisco, CA USA; 2 Memorial Sloan Kettering Cancer Center, New York, NY USA; 3 Department of General Internal Medicine, University of California, San Francisco, San Francisco, CA USA

## INTRODUCTION

The prevalence of cannabis use is rising among the US population.^1^ As cannabis continues to be legalized throughout the USA, people are turning to the internet and social media for information about its potential health benefits.^2,3^ In this study, we characterize internet claims about the health benefits of cannabis use in the lay press and evaluate the evidence base supporting those claims.

## METHODS

We performed a cross-sectional study of internet claims focused on the health benefits of cannabis use. We extracted information on claims from two different sources on June 15, 2019: (1) We searched Google for “marijuana benefits,” “weed benefits,” and “marijuana health.” Our sample includes the top ten lay webpages from each Google search. (2) We searched Buzzsumo, a social media analyzer tool that calculates online engagement with news articles, to measure each article’s engagement by its number of likes, shares, and comments on social media sites. We used the terms: “marijuana benefits OR cannabis benefits OR weed benefits,” and “marijuana health,” restricting our search to articles published in the previous 2 years (2017–2019). We excluded articles irrelevant to the potential health benefits of cannabis use, and only included high-impact articles (over 10,000 engagements) because they had the most reach with an online audience. Internet links to the scientific literature were not included since the focus of this analysis was to characterize information available to the public in the lay press. Two reviewers (NL, MG) independently reviewed webpages and articles to extract and categorize claims about the health benefits of cannabis use and tally the frequency of each claim category. Two investigators with expertise in cannabis evidence review (SK, DK)^4,5^ evaluated the literature published before November 2019 to determine claim validity based on available evidence. The two investigators searched Medline to first identify published systematic reviews. For claims with no relevant systematic reviews, randomized controlled trials were sought. Health claims were compared to the existing trial evidence and categorized as not true, partly true, and true. Disagreements were resolved by discussion.

## RESULTS

**Figure 1:**
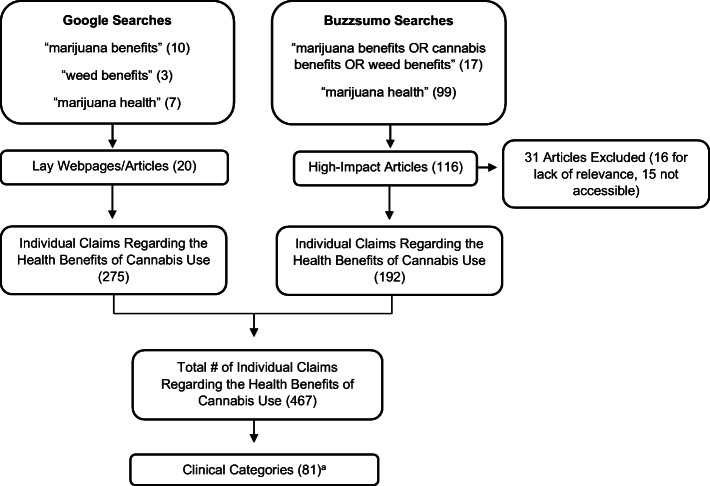
Sample construction. ^a^81 Clinical categories: general pain, cancer pain, nerve pain, migraines, fibromyalgia, epilepsy/seizures, anti-nausea/vomiting from chemotherapy, anti-nausea, cancer treatment, anxiety, multiple sclerosis, reduce muscle spasms, sleep, Alzheimer’s disease, alternative to opioids, reducing opioid dependence, neurogenesis, neuroprotective, appetite stimulant (general), eating disorders, appetite stimulant for people w/ AIDS, cachexia, improves weight loss for cancer patients, obesity, Crohn’s disease, ulcerative colitis, irritable bowel syndrome, glaucoma, depression, post-traumatic stress disorder, schizophrenia, obsessive compulsive disorder, ADHD, emotional, mood, and cognitive regulation, phobias, other mental health/mood disorders, dementia, autism spectrum disorder, leukemia, brain cancer, breast cancer, bladder cancer, lung cancer, pancreatic cancer, colon cancer, prostate cancer, skin cancer, treating movement disorders (general), amyotrophic lateral sclerosis, Parkinson’s disease, Tourette’s syndrome, respiratory, asthma, cardiovascular, blood sugar, chronic heart failure, hypertension, heart attack, stroke, treating alcoholism, quitting smoking, treating marijuana abuse, quitting other drugs, anti-inflammatory, arthritis, alcohol, sexual health, sexually transmitted diseases, female reproductive health, skin health, diabetes, hepatitis C, lupus, liver, malaria, bones, cartilage, degenerative disc disease, immune system, spinal cord injury, palliative care, digestive function.

The Google search produced 20 unique lay webpages/articles, and the Buzzsumo search produced 116 high-impact articles. We excluded 16 articles for irrelevancy (e.g., focus on cannabis policies instead of use) and 15 for inaccessibility (expired webpages), leading to 105 total sources. We found 275 individual claims regarding the health benefits of cannabis use in Google sources, and 192 claims in Buzzsumo articles. The 467 individual claims in our sample comprised 81 distinct clinical categories (Fig. 1). Of the 81 categories, 65 (80.2%) were not true, 7 (8.6%) were partly true, and 4 (4.9%) were true; 5 (6.2%) were unable to be assessed due to being too broad or vague (e.g., anti-inflammatory or digestive function). Table 1 summarizes the 10 most common categories of claims. Claims regarding the benefit of cannabis in the treatment of pain were the most common. Other common claims included the efficacy of cannabis for glaucoma, depression, nausea, muscle spasms, Parkinson’s disease, and cancer therapy, and as an alternative to opioids or reducing opioid dependence. Claims classified as “Not true” related to general pain, cancer, anxiety, post-traumatic stress disorder, neuroprotection, and Alzheimer’s disease. The remainder of claims (among the top 10 common) were true (treatment of chemotherapy-induced nausea/vomiting and spasticity from multiple sclerosis) or partly true (treatment of seizures and sleep) (Table 1).

**Table 1:** Top 10 Most Common Clinical Categories of Claims, Examples of Individual Claims Related to that Category, and Review of Trial Evidence

Clinical category	# of claims in sample (*N* = 467)	Validity of claim based on trial evidence	Availability of trial evidence/comments
General pain—treats pain, effective at relieving chronic pain, relieve pain from inflammation, pain management for back injuries, may help with bladder pain, helps treat severe pain or post-surgical pain	37 (7.9%)	Not true	-Mixed or insufficient trial evidence.^a,b,c,d,e,f^ -There is no consistent evidence that cannabis use is useful for general pain and musculoskeletal pain. Most studies of benefit are in patients with neuropathic pain.
Epilepsy/seizures—treats epilepsy, lowers seizure frequency in epilepsy patients, treats Lennox-Gastaut syndrome/Dravet syndrome	33 (7.1%)	Partly true	-Mixed or insufficient trial evidence.^c,f,g,h^ -There is trial evidence to support use of cannabinoids in refractory epilepsy of children; however, there is no trial evidence for treatment of epilepsy in adults.
Anti-nausea/vomiting from chemotherapy—oral cannabinoids and smoked cannabis can be effective against nausea and vomiting caused by chemotherapy	22 (4.7%)	True	-Preponderance of trial evidence supports the use of cannabinoid pharmaceuticals in the management of nausea and vomiting of chemotherapy.^c,d,e,l^ There are no high-quality plant-based trial examining the effectiveness of cannabis for the treatment of nausea and vomiting associated with chemotherapy.
Cancer treatment—slows spread of several types of cancer, stops cancer from spreading (at least in cell cultures), slows tumor growth, helps kill cancer cells	21 (4.5%)	Not true	-No trial evidence available.^c,i,j^ Animal and preclinical studies have suggested that cannabis may have anti-tumor properties.
Anxiety—may reduce anxiety, may relieve symptoms of social anxiety	20 (4.3%)	Not true	- There is no trial evidence supporting the use of cannabinoid pharmaceutical in generalized anxiety disorder.^c,d,k,l,m,n^ -Evidence around social anxiety is mixed. Some trials improve symptoms some trials make it worse.
Multiple sclerosis—decreases spasticity associated with multiple sclerosis, can stop negative neurological effects and muscle spasms caused by multiple sclerosis	19 (4.1%)	True	-Preponderance of trial evidence supporting claim.^c,d,e,h,o,p^ The strongest evidence available pertains to oral cannabis extract (cannabidiol or CBD), tetrahydrocannabinol (THC), and Nabiximols (combination of THC and CBD) for the treatment of the pain and spasticity associated with multiple sclerosis.
Post-traumatic stress disorder—relieves post-traumatic stress disorder symptoms, can help eliminate nightmares associated with post-traumatic stress disorder	18 (3.9%)	Not true	-No trial evidence.^k,m,n,q,r^ -Cannabis use may negatively affect post-traumatic stress disorder management.
Sleep—helps with insomnia, promotes sleep, can improve sleep quality, helps eliminate nightmares	15 (3.2%)	Partly true	-Mixed or insufficient trial evidence.^c,d,s^ -Mixed trial evidence for a variety oral plant-based extracts and pharmaceuticals. More evidence needs to establish efficacy for this indication. Some evidence that THC may have long-term harms.
Neuroprotective—can prevent brain damage after strokes and trauma, may protect brain from concussions/trauma, protects against degenerative neurological disorders	15 (3.2%)	Not true	-No trial evidence.^c,p^ Only observational studies showing an association.
Alzheimer’s disease—used to treat Alzheimer’s, may slow progression, treats neuropsychiatric symptoms of Alzheimer’s including agitation, anxiety and psychosis	13 (2.8%)	Not true	-Mixed or insufficient trial evidence.^m,t,u,v^ -Few randomized controlled trials, many with high risk of bias. Better quality studies have been negative.

^a^Rabgay K, Waranuch N, Chaiyakunapruk N, et al. The Effects of Cannabis, Cannabinoids, and Their Administration Routes on Pain Control Efficacy and Safety: A Systematic Review and Network Meta-analysis. Journal of American Pharmacists Association. 2019; 60(1): 225–234. 10.1016/j.japh.2019.07.015. ^b^Nugent SM, Morasco BJ, O’Neil ME, et al. The Effects of Cannabis Among Adults with Chronic Pain and an Overview of General Harms: A Systematic Review. *Ann Intern Med*. 2017;167(5):319–331. 10.7326/M17-0155. ^c^National Academies of Sciences, Engineering, and Medicine. The Health Effects of Cannabis and Cannabinoids: The Current State of Evidence and Recommendations for Research. *Washington, DC: The National Academies Press.* 2017. 10.17226/24625. ^d^Whiting PF, Wolff RF, Deshpande S, et al. Cannabinoids for Medical Use: A Systematic Review and Meta-analysis. *JAMA*. 2015;313(24):2456–2473. 10.1001/jama.2015.6358. ^e^Allan GM, Finley CR, Ton J, et al. Systematic Review of Systematic Reviews for Medical Cannabinoids. *Canadian Family Physician*. Published February 2018. Accessed October 29, 2019. https://www.cfp.ca/content/cfp/64/2/e78.full.pdf. ^f^Stockings E, Campbell G, Hall WD, et al. Cannabis and Cannabinoids for the Treatment of People with Chronic Noncancer Pain Conditions: A Systematic Review and Meta-Analysis of Controlled and Observational Studies. *Pain*. 2018;159(10):1932–1954. 10.1097/j.pain.0000000000001293. ^g^Gloss D & Vickrey B. Cannabinoids for epilepsy. *Cochrane Database Syst Rev*. 2014;5(3):CD009270. 10.1002/14651858.CD009270.pub3. ^h^Koppel BS, Brust JC, Fife T, et al. Systematic review: Efficacy and Safety of Medical Marijuana in Selected Neurologic Disorders: Report of the Guideline Development Subcommittee of the American Academy of Neurology. *Neurology*. 2014;82(17):1556–1563. doi.10.1212/WNL.0000000000000363. ^i^Wilkie G, Sakr B, Rizack T. Medical Marijuana Use in Oncology: A Review. JAMA Oncol. 2016;2(5):670–675. 10.1001/jamaoncol.2016.0155. ^j^Rocha FCM, dos Santos Jr. JG, Stefano SC, et al. Systematic Review of the Literature on Clinical and Experimental Trials on the Antitumor Effects of Cannabinoids in Gliomas. *Journal of Neuro-Oncology*. 2014;116(1):11–24. 10.1007/s11060-013-1277-1. ^k^Botsford S, Yang S, George TP. Cannabis and Cannabinoids in Mood and Anxiety Disorders: Impact on Illness Onset and Course, and Assessment of Therapeutic Potential. *The American Journal on Addictions*. 2019; 1–18. 10.1111/ajad.12963. ^l^Mandolini GM, Lazzaretti M, Pigoni A, et al. Pharmacological Properties of Cannabidiol in the Treatment of Psychiatric Disorders: A Critical Overview. *Epidemiology and Psychiatric Sciences*. 2018;27(4):327–335. 10.1017/S2045796018000239. ^m^Hoch E, Niemann D, von Kepper R, et al. How Effective and Safe is Medical Cannabis as a Treatment of Mental Disorders? A Systematic Review. *European Archives of Psychiatry and Clinical Neuroscience*. 2019;269(1):87–105. 10.1007/s00406-019-00984-4. ^n^Turna J, Patterson B, Van Ameringen M. Is Cannabis Treatment for Anxiety, Mood, and Related Disorders Ready for Prime Time? *Depression and Anxiety*. 2017;34:1007-1017. 10.1002/da.22664. ^o^Nielsen S, Germanos R, Weier M, et al. The Use of Cannabis and Cannabinoids in Treating Symptoms of Multiple Sclerosis: A Systematic Review of Reviews. *Curr Neurol Neurosci Rep*. 2018;18(2):8. 10.1007/s11910-018-0814-x. ^p^Kluger B, Triolo P, Jones W, et al. The Therapeutic Potential of Cannabinoids for Movement Disorders. *Mov Disord*. 2015;30(3): 313–327. 10.1002/mds.26142. ^q^O'Neil ME, Nugent SM, Morasco BJ, et al. Benefits and Harms of Plant-Based Cannabis for Posttraumatic Stress Disorder: A Systematic Review. *Ann Intern Med*. 2017;167:332–340. 10.7326/M17-0477. ^r^Walsh Z, Gonzalez R, Crosby K, et al. Medical Cannabis and Mental Health: A Guided Systematic Review. *Clinical Psychology Review*. 2017;51:15–29. 10.1016/j.cpr.2016.10.002. ^s^Gates PJ, Albertella L, Copeland J. The Effects of Cannabinoid Administration on Sleep: A Systematic Review of Human Studies. Sleep Medicine Reviews. 2014;18(6):477–487. 10.1016/j.smrv.2014.02.005. ^t^Hillen JB, Soulsby N, Alderman C, et al. Safety and effectiveness of cannabinoids for the treatment of neuropsychiatric symptoms in dementia: a systematic review. *Therapeutic Advances in Drug Safety.* 2019;10:1–23. 10.1177/2042098619846993. ^u^Krishnan S, Cairns R, Howard R. Cannabinoids for the treatment of dementia. *Cochrane Database of Systematic Reviews*. 2009. 10.1002/14651858.CD007204.pub2. ^v^Santibanez A, Sepehry AA, Hsiung G-YR. Cannabis and Alzheimer’s Disease: A Systematic Review of the evidence. *Alzheimer’s & Dementia: The Journal of the Alzheimer’s Association*. 2017;13(7). 10.1016/j.jalz.2017.06.674.

## DISCUSSION

We found that less than 5% of the internet claims about the health benefits of cannabis use were proven to be true based on available evidence. The inadequacy of the current evidence enables the proliferation of untrue claims, which inform the current social discourse on the health benefits of cannabis. More studies on the health effects of cannabis are needed to better inform the public and health care providers. Patients and providers should be cautious consumers of health information on the internet given the current state of the evidence and proliferation of false claims.
